# Identification of Genomic Variants Associated with the Risk of Acute Lymphoblastic Leukemia in Native Americans from Brazilian Amazonia

**DOI:** 10.3390/jpm12060856

**Published:** 2022-05-25

**Authors:** Luciana P. C. Leitão, Darlen C. de Carvalho, Juliana C. G. Rodrigues, Marianne R. Fernandes, Alayde V. Wanderley, Lui W. M. S. Vinagre, Natasha M. da Silva, Lucas F. Pastana, Laura P. A. Gellen, Matheus C. E. Assunção, Sweny S. M. Fernandes, Esdras E. B. Pereira, André M. Ribeiro-Dos-Santos, João F. Guerreiro, Ândrea Ribeiro-dos-Santos, Paulo P. de Assumpção, Sidney E. B. dos Santos, Ney P. C. dos Santos

**Affiliations:** 1 Oncology Research Nucleus, Universidade Federal do Pará, Belém 66073-005, PA, Brazil; colaresluciana@gmail.com (L.P.C.L.); darlen.c.carvalho@gmail.com (D.C.d.C.); julianacgrodrigues@gmail.com (J.C.G.R.); fernandesmr@yahoo.com.br (M.R.F.); luivinagre@gmail.com (L.W.M.S.V.); ntshmonte@gmail.com (N.M.d.S.); lucas.favacho.celular@gmail.com (L.F.P.); laura.patricia.agellen@hotmail.com (L.P.A.G.); matheus.assuncao@icb.ufpa.br (M.C.E.A.); assumpcaopp@gmail.com (P.P.d.A.); 2 Faculdade de Ciências Médicas do Pará (FACIMPA), Marabá 68508-030, PA, Brazil; 3 Pediatrics Department, Ophir Loyola Hospital, Belém 66063-240, PA, Brazil; alaydevieira@yahoo.com.br (A.V.W.); swenymf@gmail.com (S.S.M.F.); 4 Human and Medical Genetics Laboratory, Instituto de Ciências Biológicas, Universidade Federal do Pará, Belém 66075-110, PA, Brazil; esdrasedgarbp@gmail.com (E.E.B.P.); andremrsantos@gmail.com (A.M.R.-D.-S.); joao.guerreiro53@gmail.com (J.F.G.); akely@ufpa.br (Â.R.-d.-S.); sidneysantos@ufpa.br (S.E.B.d.S.); 5 Instituto Tocantinense Presidente Antônio Carlos (ITPAC), Abaetetuba 68440-000, PA, Brazil

**Keywords:** Acute Lymphoblastic Leukemia, Amerindian populations, genetic susceptibility

## Abstract

A number of genomic variants related to native American ancestry may be associated with an increased risk of developing Acute Lymphoblastic Leukemia (ALL), which means that Latin American and hispanic populations from the New World may be relatively susceptible to this disease. However, there has not yet been any comprehensive investigation of the variants associated with susceptibility to ALL in traditional Amerindian populations from Brazilian Amazonia. We investigated the exomes of the 18 principal genes associated with susceptibility to ALL in samples of 64 Amerindians from this region, including cancer-free individuals and patients with ALL. We compared the findings with the data on populations representing five continents available in the 1000 Genomes database. The variation in the allele frequencies found between the different groups was evaluated using Fisher’s exact test. The analyses of the exomes of the Brazilian Amerindians identified 125 variants, seven of which were new. The comparison of the allele frequencies between the two Amerindian groups analyzed in the present study (ALL patients vs. cancer-free individuals) identified six variants (rs11515, rs2765997, rs1053454, rs8068981, rs3764342, and rs2304465) that may be associated with susceptibility to ALL. These findings contribute to the identification of genetic variants that represent a potential risk for ALL in Amazonian Amerindian populations and might favor precision oncology measures.

## 1. Introduction

Acute Lymphoblastic Leukemia (ALL) is the most common cancer in children and is the principal cause of childhood mortality due to malignant disease [1,2]. The genetic etiology of ALL is driven by an ample diversity of alterations of the pathways responsible for the regulation of the cell cycle in the lymphoid precursors of the B and T cell lines, which include chromosomal translocations, mutations, and aneuploidy [3,4,5]. In recent years, Genome-Wide Association Studies (GWASs) have identified a number of loci associated with the risk of developing ALL, including the *ARID5B*, *IKZF1*, *PIP4K2A*, *CEBPE*, *GATA3*, *BMI*, and *CDKN2A* genes [6,7,8,9,10,11]. 

Most GWASs addressing ALL susceptibility have focused on homogeneous populations in regions such as Europe or North America, whereas the highest incidence of childhood ALL is found in populations with a major component of native American ancestry, such as the Latin American and hispanic populations of the New World [9,12,13,14]. These populations are highly admixed and, like the population of Brazil, are primarily descended from European, African, and native American ancestors [15,16], and the high incidence of ALL observed in Latin American and hispanic populations has been attributed [9,12,13,17] to genetic risk factors related to native American ancestry. Despite the evidence that genetic variants related to native American ancestry may influence the incidence of childhood ALL, no data are available on the distribution of these variants in traditional Amerindian populations.

Given this, in the present study, we investigated the genetic variants potentially involved in the etiology of ALL in traditional Amerindian populations from Brazilian Amazonia. For this, we used New Generation Sequencing (NGS) to define the exomes of 17 genes associated with susceptibility to ALL in samples obtained from indigenous groups that inhabit Brazilian Amazonia, in both ALL patients and cancer-free individuals. The variants encountered in this initial analysis were compared with the data available on populations representing five different continents, which were obtained from the 1000 Genomes database.

## 2. Materials and Methods

### 2.1. Ethics, Consent, and Permissions

The present study was approved by Brazilian National Committee on Research Ethics—CONEP (identified by No 1062/2006 and 123/1998). All participants signed a free-informed consent as well as the tribe leaders and when necessary, a translator explained the project and the importance of the research. Their materials were collected according to the Declaration of Helsinki.

### 2.2. Study Population

The study sample included 59 cancer-free Amerindians and five individuals diagnosed with ALL. All the participants were members of isolated ethnic groups located in Brazilian Amazonia (Table S1). The genomic Amerindian ancestry of all these individuals was quantified and found to be at least 64% in all cases. The Amerindians with ALL were diagnosed and treated in two public hospitals specialized in the treatment of childhood cancer, the Ophir Loyola Hospital and the Octavio Lobo Childhood Oncology Hospital, both located in the city of Belém, in Pará state, northern Brazil. The clinical and demographic data on these patients are presented in Table S2. 

Data were obtained from the 1000 Genomes Project database (available at https://www.1000genomes.org, accessed on: 20 February 2021) to provide comparisons with ethnic groups from other continents. This sample included 661 individuals from Africa (AFR), 503 from Europe (EUR), 347 from the Americas (AMR), 504 from East Asia (EAS), and 489 from South Asia (SAS).

### 2.3. Selection of the Genes

A total of 17 genes were selected for the present study (see Table S3). The genes were selected based on a search of the literature available in the NCBI and Ensembl databases, and on GWASs or studies of other genetic markers associated with the risk of ALL. More details are described in Table S4.

### 2.4. Extraction of the DNA and Preparation of the Exomes

Samples of 5 mL of peripheral blood were collected from each of the participants of the study. The genetic material was extracted from these blood samples using the Roche Applied Science DNA extraction kit (Roche, Penzberg, Germany) following the manufacturer’s instructions, and it was quantified using a NanoDrop 1000 spectrophotometer (NanoDrop Technologies, Wilmington, DE, USA). The exome libraries were prepared using the commercial Nextera Rapid Capture Exome kit (Illumina, San Diego, CA, EUA) and the SureSelect Human All Exon V6 kit (Agilent, Santa Clara, CA, USA), with the manufacturer’s protocol being followed in both cases. The sequencing reactions were run in the NextSeq 500^®^ platform (Illumina^®^, San Diego, CA, USA) using the NextSeq 500 high-output v2 300 cycle kit (Illumina^®^, San Diego, CA, USA). 

### 2.5. Bioinformatic Analysis

The bioinformatic analyses followed the approach described by Ribeiro-Dos-Santos et al. [18] and Rodrigues et al. [19]. For this, the sequences were first filtered to eliminate low-quality reads, and then mapped and aligned with the reference genome (GRCh38) using BWA v.0.7. The alignment was then processed to remove duplicate sequences, recalibrate the mapping quality, and finalize local realignment. The results were processed in GATK v.3.2 to identify the reference genome variants. The Viewer of Variants (ViVa^®^, Universidade Federal do Rio Grande do Norte, Natal, RN, Brazil) software was used to analyze the annotations of the variants. The variants were annotated in three databases—SnpEff v.4.3.T, Ensembl Variant Effect Predictor (Ensembl version 99), and ClinVar (v.2018–10). The SIFT (v.6.2.1), PolyPhen-2 (v.2.2), LRT (November, 2009), Mutation Assessor (v.3.0), Mutation Taster (v.2.0), FATHMM (v.2.3), PROVEAN (v.1.1.3), MetaSVM (v.1.0), M-CAP (v.1.4), and FATHMM-MKL databases were used for the in silico prediction of pathogenicity.

### 2.6. Statistical Analysis

For the statistical analyses, the cancer-free Amerindians were assigned to the Native (NAT) group, while the ALL patients were in the ALL_NAT (Native with ALL) group. All the analyses were run in the R v.3.5.1 program. The differences in the allelic frequencies between the NAT and ALL_NAT were evaluated using Fisher’s exact test, which was also applied to the comparisons with the continental populations. A *p* ≤ 0.05 significance level was considered for all the analyses.

## 3. Results

The analysis of the exomes of the 64 Amerindians investigated in the present study revealed the presence of 125 variants, seven of which were new. The SnpEff software [20] was used to annotate and predict the effects of these variants. This procedure classifies the impact of the variants in four categories: (i) modifier (no evidence of impact), (ii) low (no apparent alteration of protein function), (iii) moderate (some alteration of protein function), and (iv) high (high level of impact on protein function). The 118 variants identified in the 17 genes investigated in the present study are described in Table S3. Four of the new variants were classified as modifiers (Table 1), two as low effect, and one as moderate. The majority of these new variants were found in the ALL_NAT group, at frequencies of less than 0.1%.

As some of the variants were not covered adequately in one or other of the groups (NAT or ALL_NAT), they were excluded from the comparison of allele frequencies. This left 64 variants that were included in the analysis of association. The frequencies of these variants are compared between groups in Table 2.

The analyses revealed significant differences between the two groups in six variants of five genes: *PIP4K2A* (variants rs2765997 and rs1053454), *CDKN2A* (rs11515), *IGF2BP1* (rs8068981), *USP7* (rs2304465), and *WWOX* (rs3764342). The allele frequencies of these six variants recorded in the ALL_NAT group were also compared with those recorded in the 1000 Genomes Project for the five continental populations (AFR, AMR, EAS, EUR, and SAS). The comparisons are shown in Table 3 and the *p* values, in Table 4.

The analyses revealed significant differences between the Amerindian group (ALL_NAT) and all the continental populations (AFR, AMR, EAS, EUR and SAS) in the frequency of the rs11515 variant of the *CDKN2A* gene. The frequency of the rs8068981 variant of the IGF2BP1 gene was also significantly different from that of the EAS population, while the frequency of the rs2765997 variant of the *PIP4K2A* gene was significantly different from those of the AFR and EAS populations.

## 4. Discussion

The incidence of ALL is relatively high in populations with a high degree of native American ancestry, such as Latin American and Hispanic populations, which has been attributed, in part, to the contribution of molecular markers associated with a high risk of ALL in these populations [9,12,13,14,21]. Despite this known association, no genomic data are available on the susceptibility to ALL of traditional Amerindian populations from Brazilian Amazonia. In the present study, we investigated the exomes of 17 of the principal genes associated with susceptibility to ALL in samples of indigenous Amazonian populations, including ALL patients and cancer-free individuals. It would be interesting to demonstrate whether the results observed for the markers investigated here in Amerindian populations would demonstrate the same profile in a larger cohort of leukemic patients from the same ethnic group. However, samples from indigenous patients with ALL are difficult to obtain, given the rarity of the disease and due to the fact that these populations inhabit remote and difficult-to-access rural regions, which reflects on the difficulty of care and clinical follow-up of these patients.

During the study, we identified seven new variants in four genes (*IKZF1*, *IKZF3*, *WWOX*, and *ZPBP2*), most of which were present in the ALL_NAT group, that is, Amerindian ALL patients. These genes are known to have alleles associated with some level of risk in the etiology of ALL in different populations [17,22,23,24]. Given this, we believe that the new variants identified in the present study should be investigated further as potential risk factors for the incidence of ALL in Amerindian populations. 

We also identified six variants of five genes that we associated with susceptibility to ALL (*CDKN2A*_rs11515, *PIP4K2A*_rs2765997 and rs1053454, *IGF2BP1*_rs8068981, *WWOX*_ rs3764342, and *USP7*_rs2304465) given the significant differences in the frequencies recorded in the two Amerindian groups (ALL patients and cancer-free individuals), and between the ALL group and populations of other groups from around the world.

The *CDKN2A* gene plays an important role in leukemogenesis. The rs11515 variant is located in the 3′-untranslated region (UTR) of the *CDKN2A* gene, and is known to be associated with a number of different types of cancer [25], including breast cancer [26], glioblastoma [27], melanoma [28], and colorectal cancer [29]. However, no data are available on the possible influence of this variant on the risk of developing ALL. In the present study, we recorded a high frequency of this variant in the Amerindian population, which was significantly higher than that recorded in other populations around the world. This indicates that research on this variant should be prioritized for the identification of its potential role in the etiology of ALL in this population. 

A number of previous studies of genetic polymorphisms in the *PIP4K2A* gene have identified an association with susceptibility to ALL [30,31]. We identified an association with ALL in two variants (rs2765997 and rs105334534) of the *PIP4K2A* gene, given that both were more frequent in the Amerindian population, in comparison with the other continental populations, which may reflect a potential correlation with the risk of ALL. 

The *IGF2BP1* protein is expressed in a number of different types of cancer, including leukemia. In a recent study based on in vivo and in vitro analyses, Elcheva et al. [32] found evidence of a significant correlation between *IGF2BP1* and the aggressiveness of leukemia, through the persistence of tumorigenicity by increasing critical transcriptional and metabolic regulators. These authors also emphasized that the *IGF2BP1* gene is often positively regulated in many types of malignant disease, and is not expressed in most normal tissue, which means that it is a potentially important target for anticancer therapy. The results of the present study indicate that the rs8068981 variant of the *IGF2BP1* gene is present at similar frequencies in the Amerindians with ALL and the European population, but contrasts with the Asian populations.

We also identified in the Amerindian population a positive association with the rs3764342 variant of the *WWOX* gene, which has a suppressor role in solid cancers, with the loss of function resulting in alterations in the adhesion of the cancerous cells to the extra-cellular matrix, which affects cell migration and metastasis. A number of studies have contributed to the description of the role of this gene in leukemic malignancies. Luo et al. [33] found that the expression of the *WWOX* mRNA and protein is significantly reduced or absent in cases of leukemia and their cell lines in comparison with the controls, which is consistent with the findings of Cui et al. [34], who evaluated patients diagnosed with different types of leukemia. 

In the present study, the rs2304465 variant of the *USP7* gene was also more frequent in Amerindian patients. Jin et al. [35] found an association between *USP7* and a lack of ubiquitination and the stabilization of the *NOTCH1* oncogene, which contributes to the control of the cell growth of the T cells in lymphoblastic leukemia. 

## 5. Conclusions

The present study is the first to investigate the exome of genes involved in the etiology of Acute Lymphoblastic Leukemia (ALL) in Amerindian populations from the Amazon region. The results of the study provide important genetic data related to the etiology of ALL in such population in which genetic investigations are scarce. The study also contributes to the identification of variants of potential risk involved in the etiology of ALL in Amerindian populations, as well as in other Brazilian populations formed through a high level of admixture with this indigenous group.

## Figures and Tables

**Table 1:** The new gene variants identified in the Amerindian population investigated in the present study.

Chromosome	Chromosomal Position	Gene	Impact	Reference Allele	Group
chr7	50368329	*IKZF1*	MODIFIER	C	ALL_NAT
chr16	78108386	*WWOX*	MODIFIER	T	ALL_NAT
chr16	78386853	*WWOX*	LOW	A	NAT
chr17	39832190	*IKZF3*	MODIFIER	T	NAT
chr17	39765793	*IKZF3*	MODERATE	C	ALL_NAT
chr17	39868422	*ZPBP2*	MODIFIER	C	ALL_NAT
chr17	39765799	*IKZF3*	LOW	C	ALL_NAT

**Table 2:** Comparisons of the frequencies of the alleles investigated in the present study between the two Amerindian groups, that is, ALL patients (ALL_NAT) and cancer-free individuals (NAT).

		Frequency in Group:		
dbSNP	Gene	ALL_NAT	NAT	* p
rs773061413	*ARID5B*	0.20	0	0.0781
rs513349	*BAK1*	0.80	0.56	0.3868
rs11515	*CDKN2A*	0.60	1	**0.0050**
rs3088440	*CDKN2A*	0.50	0.56	1
rs974336	*CDKN2B*	0.25	0	0.0781
rs2302901	*ELK3*	0	0.03	1
rs422628	*GATA3*	0.75	0.97	0.2197
rs2305479	*GSDMB*	0.30	0.43	1
rs2305480	*GSDMB*	0.30	0.43	1
rs11078928	*GSDMB*	0.30	0.43	1
rs12450091	*GSDMB*	0.20	0.26	1
rs11078927	*GSDMB*	0.30	0.42	1
rs8068981	*IGF2BP1*	0.60	0	**0.0002**
rs62078405	*IGF2BP1*	0.20	0	0.0781
rs2289637	*IGF2BP1*	0.20	0.06	0.3434
rs10899750	*IKZF1*	0.50	0.67	0.6572
rs61731355	*IKZF1*	0.20	0.31	1
rs12669559	*IKZF1*	0.10	0.28	1
rs907092	*IKZF3*	0.20	0.04	0.2197
rs3824810	*LHPP*	0.60	0.57	1
rs3824809	*LHPP*	0.60	0.71	0.6287
rs6597801	*LHPP*	0.70	0.88	0.1907
rs943192	*PIP4K2A*	0.80	0.98	0.1513
rs2765997	*PIP4K2A*	0.50	0.96	**0.0043**
rs1132816	*PIP4K2A*	0.10	0.01	0.1774
rs61731109	*PIP4K2A*	0.10	0.01	0.1774
rs1053454	*PIP4K2A*	0.90	0	**7.264 × 10^−7^**
rs2447919	*USP7*	0.13	0.31	1
rs11551182	*USP7*	0.30	0.40	1
rs139138924	*USP7*	1	1	1
rs2304465	*USP7*	0.40	0	**0.0050**
rs1382390	*USP7*	1.00	1	1
rs2447918	*USP7*	0.16	0	0.0781
rs117832776	*WWOX*	0.20	0	0.0781
rs383362	*WWOX*	0.30	0.16	0.3052
rs77897021	*WWOX*	0.10	0.08	0.4545
rs12934985	*WWOX*	0.10	0.21	1
rs3764342	*WWOX*	0.40	0	**0.0050**
rs200461412	*WWOX*	0.20	0.03	0.2197
rs140060332	*WWOX*	0.20	0	0.0781
rs4130513	*WWOX*	0.30	0.09	0.1551
rs11545029	*WWOX*	0.10	0.08	0.4545
rs8050128	*WWOX*	0.75	0.41	0.1590
rs75559202	*WWOX*	0.10	0.09	0.4545
rs146481440	*WWOX*	0.20	0.06	0.3434
rs12446823	*WWOX*	0.10	0.01	0.1774
rs2288034	*WWOX*	0.60	0.32	0.3292
rs144601717	*WWOX*	0.10	0.01	0.1774
rs2303190	*WWOX*	0	0.22	0.5739
rs3764340	*WWOX*	0.20	0.09	0.3990
rs2303191	*WWOX*	0.80	0.98	0.1513
rs2288035	*WWOX*	0.10	0.08	0.4545
rs8048830	*WWOX*	0.25	0.01	0.1513
rs67493355	*WWOX*	0.25	0.16	1
rs202093359	*WWOX*	0.20	0.08	0.3990
rs11545028	*WWOX*	0.10	0.22	1
rs2288033	*WWOX*	0.40	0.25	0.5917
rs7199110	*WWOX*	0.60	0.40	0.6423
rs555396422	*WWOX*	0.10	0	0.0923
rs384216	*WWOX*	0	0	1
rs75027016	*ZPBP2*	0.30	0.15	0.2662
rs11557467	*ZPBP2*	0.30	0.43	1
rs11557466	*ZPBP2*	0.30	0.42	1
rs10852935	*ZPBP2*	0.30	0.43	1

* Fisher’s exact test.

**Table 3:** Allele frequencies of the six key variants identified in the present study in the ALL_NAT populations (Table 2) and the five continental populations from the 1000 Genomes Project (AFR, AMR, EAS, EUR, and SAS).

dbSNP	Gene	Frequency in the Population:					
		ALL_NAT	AFR	AMR	EAS	EUR	SAS
rs11515	*CDKN2A*	0.60	0.16	0.08	0.02	0.15	0.05
rs8068981	*IGF2BP1*	0.60	0.57	0.68	0.95	0.71	0.82
rs2765997	*PIP4K2A*	0.50	0.18	0.20	0.05	0.37	0.37
rs1053454	*PIP4K2A*	0.90	0.85	0.81	0.65	0.64	0.78
rs2304465	*USP7*	0.40	0.66	0.58	0.70	0.59	0.49
rs3764342	*WWOX*	0.40	0.55	0.14	0.30	0.11	0.32

**Table 4:** The p values recorded for the pairwise comparisons (ALL_NAT vs. AFR, AMR, EAS, EUR or SAS) of the allele frequencies of the six key variants identified in the present study (see Table 3).

dbSNP	Gene	Frequency ALL_NAT	*p* for the ALL_NAT Population Versus:				
			AFR	AMR	EAS	EUR	SAS
rs11515	*CDKN2A*	0.67	**0.0349**	**0.0050**	**0.0001**	**0.0267**	**0.0015**
rs8068981	*IGF2BP1*	0.60	1	0.6577	**0.0263**	0.6283	0.2302
rs2765997	*PIP4K2A*	0.50	**0.0452**	0.0579	**0.0015**	0.3627	0.3663
rs1053454	*PIP4K2A*	0.90	1	0.5880	0.1702	0.1655	0.5911
rs2304465	*USP7*	0.40	0.3447	0.6535	0.1602	0.6536	1
rs3764342	*WWOX*	0.40	0.6626	0.1595	0.6411	0.0960	0.6550

## Data Availability

Not applicable.

## Supplementary Materials

The following supporting information can be downloaded at: https://www.mdpi.com/article/10.3390/jpm12060856/s1, Table S1. Names and the number of individuals in each population group analyzed in the present study; Table S2. Epidemiological and clinical characteristics of the patients included in the study; Table S3. Description of the variants found in the Amerindian population investigated in the present study; Table S4 [36,37,38,39,40,41,42,43,44,45,46,47,48]. Description of the studies selected to choose the genes studied.
