# Supplementary material for: Identification of Genomic Variants Associated with the Risk of Acute Lymphoblastic Leukemia in Native Americans from Brazilian Amazonia
Source: J Pers Med. 2022 May 25;12(6):856. doi: 10.3390/jpm12060856 (PMC9224820; doi:10.3390/jpm12060856)
Supplement: Supplementary file 1 [file jpm-12-00856-s001.zip › jpm-1664101-supplementary.pdf]

Table S1. Names and the number of individuals in each population group analyzed in the present study.

Acronym	Populations	Number of individuals
AKW	Asurini do Koatinemo	5
ARA	Arara/Arara do Iriri*	7
ARW	Araweté	6
AST	Asurini do Trocará	16
AWA	Awa-Guajá	7
KAY	Kayapó	1
ODJ	Xikrin Odjá	2
PTJ	Zo'é	5
WPI	Wayãpy	5
XIK	Xikrin do Cateté	6
CAR	Caripunas*	2
JUR	Jurunas*	2

* Patients with indigenous ALL are from the following groups: two from the Caripunas group, two from the Jurunas group, and one from the Arara group.

Table S2. Epidemiological and clinical characteristics of the patients included in the study.

Variable	n (%)
Sex	
Male	4 (80.0)
Female	1 (20.0)
Mean age at diagnosis (years, SD ±)	4.6±2.5
Leukometry at diagnosis (μL)	
<50.000	5 (100)
≥50.000	0
Risk group	
Standard	0
Medium	0
High	5 (100)
Cell type	
Cell B	4 (80.0)
Cell T	1 (20.0)
Treatment protocol	
BFM-2002	5 (100)

Abbreviations: SD, Standard Deviation.

Table S3. Description of the variants found in the Amerindian population investigated in the present study.

Chromosome	Chromosomal Position	dbSNP	Gene	Impact	Reference Allele
chr6	33573942	rs513349	*BAK1*	MODIFIER	A
chr7	50391707	rs78889803	*IKZF1*	MODIFIER	G
chr7	50368335	rs10899750	*IKZF1*	MODIFIER	A
chr7	50400069	rs61731355	*IKZF1*	LOW	C
chr7	50368129	rs1490393465	*IKZF1*	MODIFIER	G
chr7	50327957	rs1035961399	*IKZF1*	MODIFIER	G
chr7	50368079	rs12669559	*IKZF1*	MODIFIER	T
chr9	21974523	rs372058542	*CDKN2A*	MODIFIER	G
chr9	22006274	rs2069426	*CDKN2B*	MODIFIER	G
chr9	21970952	rs758832729	*CDKN2A*	MODERATE	C
chr9	21968200	rs11515	*CDKN2A*	MODIFIER	C
chr9	21968292	rs769718733	*CDKN2A*	MODIFIER	G
chr9	22006349	rs974336	*CDKN2B*	MODIFIER	C
chr9	21968160	rs3088440	*CDKN2A*	MODIFIER	G
chr10	61902621	rs147151840	*ARID5B*	MODIFIER	G
chr10	1,24E+08	rs201971378	*LHPP*	MODERATE	C
chr10	8069446	rs422628	*GATA3*	MODIFIER	C
chr10	1,24E+08	rs141786334	*LHPP*	MODIFIER	T
chr10	8073705	rs373223232	*GATA3*	MODIFIER	TA
chr10	1,24E+08	rs75426652	*LHPP*	MODERATE	C
chr10	22541790	rs943192	*PIP4K2A*	MODIFIER	G
chr10	62000328	rs773061413	*ARID5B*	MODIFIER	CT
chr10	61940162	rs79274707	*ARID5B*	MODIFIER	T
chr10	62091275	rs117297247	*ARID5B*	LOW	G
chr10	1,24E+08	rs74160914	*LHPP*	MODIFIER	G
chr10	22573353	rs10508649	*PIP4K2A*	LOW	T
chr10	22607850	rs2765997	*PIP4K2A*	MODIFIER	C
chr10	8073705	rs751198886	*GATA3*	MODIFIER	TAA
chr10	22714297	rs1132816	*PIP4K2A*	LOW	A
chr10	1,24E+08	rs3824810	*LHPP*	MODIFIER	A
chr10	1,24E+08	rs148437146	*LHPP*	MODIFIER	GC
chr10	22541913	rs61731109	*PIP4K2A*	LOW	C
chr10	8058669	rs2228254	*GATA3*	LOW	T
chr10	22537164	rs1053454	*PIP4K2A*	MODIFIER	A
chr10	22537249	rs80142698	*PIP4K2A*	LOW	T
chr10	61902144	rs56102370	*ARID5B*	MODIFIER	T
chr10	22541928	rs62640376	*PIP4K2A*	LOW	G
chr10	22550699	rs2230469	*PIP4K2A*	MODERATE	T
chr10	1,24E+08	rs3824809	*LHPP*	MODIFIER	C
chr10	22326393	rs79786472	*BMI1*	MODIFIER	G
chr10	1,24E+08	rs146909161	*LHPP*	LOW	C
chr10	1,24E+08	rs146909161	*LHPP*	HIGH	C
chr10	1,24E+08	rs6597801	*LHPP*	MODERATE	A
chr12	96259723	rs4762144	*ELK3*	LOW	C
chr12	96247238	rs35332676	*ELK3*	MODERATE	C
chr12	96267236	rs2302901	*ELK3*	MODIFIER	G
chr14	23119111	rs78537674	*CEBPE*	MODIFIER	C
chr16	79211549	rs117832776	*WWOX*	MODIFIER	C

chr16	79211923	rs383362	*WWOX*	MODIFIER	G
chr16	79211889	rs77897021	*WWOX*	MODIFIER	A
chr16	78115170	rs12934985	*WWOX*	MODIFIER	A
chr16	78432861	rs3764342	*WWOX*	MODIFIER	A
chr16	78432637	rs73572838	*WWOX*	MODERATE	G
chr16	8910707	rs2447916	*USP7*	MODIFIER	C
chr16	8916969	rs34357840	*USP7*	MODIFIER	T
chr16	79211692	rs200461412	*WWOX*	MODERATE	C
chr16	79211799	rs140060332	*WWOX*	MODIFIER	G
chr16	79211466	rs16949964	*WWOX*	MODIFIER	C
chr16	78114933	rs12934051	*WWOX*	MODIFIER	C
chr16	78424853	rs4130513	*WWOX*	MODIFIER	G
chr16	8916969	rs878958458	*USP7*	MODIFIER	TA
chr16	78386878	rs11545029	*WWOX*	MODERATE	G
chr16	8900706	rs2447919	*USP7*	MODIFIER	C
chr16	8895546	rs1677473	*USP7*	MODIFIER	A
chr16	8905281	rs11551182	*USP7*	LOW	T
chr16	8923204	rs139138924	*USP7*	MODIFIER	T
chr16	78278600	rs8050128	*WWOX*	MODIFIER	C
chr16	8898485	rs2304465	*USP7*	MODIFIER	G
chr16	78425018	rs75559202	*WWOX*	MODERATE	C
chr16	79211849	rs146481440	*WWOX*	MODIFIER	C
chr16	78756880	rs73573724	*WWOX*	MODIFIER	G
chr16	78386890	rs74944733	*WWOX*	MODERATE	G
chr16	78386985	rs12446823	*WWOX*	MODIFIER	C
chr16	78386977	rs748827826	*WWOX*	MODIFIER	C
chr16	78108410	rs149533117	*WWOX*	MODIFIER	TG
chr16	8936587	rs1382390	*USP7*	MODIFIER	C
chr16	79211893	rs142218559	*WWOX*	MODIFIER	A
chr16	8916589	rs57510291	*USP7*	MODIFIER	A
chr16	78424910	rs7201683	*WWOX*	MODERATE	C
chr16	78164360	rs76347249	*WWOX*	MODIFIER	C
chr16	8902520	rs2447918	*USP7*	MODIFIER	C
chr16	79212059	rs2288034	*WWOX*	MODIFIER	C
chr16	78115038	rs144601717	*WWOX*	MODERATE	C
chr16	78164339	rs2303190	*WWOX*	MODIFIER	G
chr16	8915641	rs79028709	*USP7*	MODIFIER	T
chr16	78432540	rs3764340	*WWOX*	MODERATE	C
chr16	78164295	rs2303191	*WWOX*	LOW	T
chr16	8920493	rs1677470	*USP7*	MODIFIER	T
chr16	78278630	rs77067228	*WWOX*	MODIFIER	A
chr16	79212033	rs2288035	*WWOX*	MODIFIER	G
chr16	78278592	rs8048830	*WWOX*	MODIFIER	G
chr16	78108411	rs67493355	*WWOX*	MODIFIER	G
chr16	79211576	rs202093359	*WWOX*	MODIFIER	A
chr16	8894721	rs111792557	*USP7*	MODIFIER	G
chr16	78099774	rs11545028	*WWOX*	MODIFIER	C
chr16	79212064	rs2288033	*WWOX*	MODIFIER	T
chr16	78756868	rs7199110	*WWOX*	MODIFIER	C
chr16	78100053	rs555396422	*WWOX*	MODIFIER	G
chr16	79211568	rs384216	*WWOX*	MODIFIER	T
chr17	39905964	rs2305479	*GSDMB*	MODERATE	C
chr17	49045163	rs8068981	*IGF2BP1*	MODIFIER	T

chr17	49044189	rs62078405	*IGF2BP1*	MODIFIER	G
chr17	39909171	rs9909282	*GSDMB*	MODIFIER	C
chr17	39868586	rs75027016	*ZPBP2*	LOW	C
chr17	39872381	rs11557467	*ZPBP2*	MODERATE	G
chr17	48999087	rs369408885	*IGF2BP1*	MODIFIER	A
chr17	39864166	rs1453559	*IKZF3*	MODIFIER	T
chr17	39905943	rs2305480	*GSDMB*	MODERATE	G
chr17	48999189	rs2411760	*IGF2BP1*	MODIFIER	G
chr17	39868373	rs11557466	*ZPBP2*	LOW	C
chr17	39908216	rs11078928	*GSDMB*	HIGH	T
chr17	49042470	rs2289637	*IGF2BP1*	MODIFIER	C
chr17	48999101	rs117838533	*IGF2BP1*	LOW	A
chr17	39766006	rs907092	*IKZF3*	LOW	G
chr17	39912368	rs12450091	*GSDMB*	MODERATE	T
chr17	39875421	rs10852935	*ZPBP2*	LOW	C
chr17	39908152	rs11078927	*GSDMB*	MODIFIER	C
chr17	48999087	rs770215863	*IGF2BP1*	MODIFIER	AT

Table S4. Description of the studies selected to choose the genes studied.

Gene	Population Studied	Sample Number	Citation
ARID5B	China	1466 non-ALL and 466 B lineage ALL	Hao et al. [36]
	Multicentric	308 cases and 6661 non-ALL control	Perez-Andreu et al. [37]
	European ancestry	969 cases and 2365 control	Papaemmanuil et al. [6]
	Japan	527 case and 3882 control	Urayama et al. [38]
BAK1	European ancestry	959 cases and 2624 control	Semmes et al. [39].
	Multiethnic Center	2237 cases and 3461 control	Vijayakrishnan et al. [40]
BMI1	United States	542 cases and 1192 controls	Brown et al. [41]
CDKN2A	United States and Germany	1979 cases and 10,784 cases	Vijayakrishnan et al. [11]
	United Kingdom	2386 cases and 2419 controls	Sherborne et al. [42]
	Multiethnic	1605 cases and 6661 control	Xu et al. [9]
CDKN2B	Europe	1210 cases and 4144 control	Hungate et al. [43]
	Multiethnic	1605 cases and 6661 control	Xu et al. [9]
CEBPE	China	451 cases and two control groups (in house = 456 and public = 10640)	Liao et al. [44]
	Yemen	136 cases and 153 control	Al-Absi et al. [45]
ELK3	United Kingdom and Germany	1658 cases and 7224 control	Vijayakrishnan et al. [46]
GATA3	United States	2597 new cases and 491 of validation	Zhang et al. [47]
	United States	542 cases and 1192 controls	Brown et al. [41]
	Chine	1466 non-ALL and 466 B lineage ALL	Hao et al. [36]
IGF2BP1	Multiethnic Center	2237 cases and 3461 control	Vijayakrishnan et al. [40]
LHPP	United Kingdom and Germany	1658 cases and 7224 control	Vijayakrishnan et al. [46]
IKZF1	United States	542 cases and 1192 controls	Brown et al. [41]

	China	451 cases and two control groups (in house = 456 and public = 10640)	Liao et al. [16]
	Japan	527 case and 3882 control	Urayama et al. [38]
PIP4K2A	Japan	527 case and 3882 control	Urayama et al. [38]
	China	451 cases and two control groups (in house = 456 and public = 10640)	Liao et al. [44]
USP7	United States	1191 cases and 12178 control	Qian et al. [48]
WWOX	China	1184 cases and 3219 controls	Shi et al. [24]
IKZF3	Latin / non-Latino white / African American	3263 cases and 15977 controls	Wiemels et al. [17]
ZPBP2	Latin / non-Latino white / African American	3263 cases and 15977 controls	Wiemels et al. [17]
GSDMB	Latin / non-Latino white / African American	3263 cases and 15977 controls	Wiemels et al. [17]
